# Development and use of iron oxide nanoparticles (Part 1): Synthesis of iron oxide nanoparticles for MRI

**DOI:** 10.2349/biij.6.2.e12

**Published:** 2010-04-01

**Authors:** J Lodhia, G Mandarano, NJ Ferris, P Eu, SF Cowell

**Affiliations:** 1 Division of Medical Radiations, School of Medical Sciences, RMIT University, Victoria, Australia; 2 Peter MacCallum Cancer Institute, Melbourne, Victoria, Australia

**Keywords:** Magnetic resonance imaging (MRI), iron oxide, nanoparticles, synthesis

## Abstract

Contrast agents, such as iron oxide, enhance MR images by altering the relaxation times of tissues in which the agent is present. They can also be used to label targeted molecular imaging probes. Unfortunately, no molecular imaging probe is currently available on the clinical MRI market. A promising platform for MRI contrast agent development is nanotechnology, where superparamagnetic iron oxide nanoparticles (SPIONS) are tailored for MR contrast enhancement, and/or for molecular imaging. SPIONs can be produced using a range of methods and the choice of method will be influenced by the characteristics most important for a particular application. In addition, the ability to attach molecular markers to SPIONS heralds their application in molecular imaging.

There are many reviews on SPION synthesis for MRI; however, these tend to be targeted to a chemistry audience. The development of MRI contrast agents attracts experienced researchers from many fields including some researchers with little knowledge of medical imaging or MRI. This situation presents medical radiation practitioners with opportunities for involvement, collaboration or leadership in research depending on their level of commitment and their ability to learn. Medical radiation practitioners already possess a large portion of the understanding, knowledge and skills necessary for involvement in MRI development and molecular imaging. Their expertise in imaging technology, patient care and radiation safety provides them with skills that are directly applicable to research on the development and application of SPIONs and MRI.

In this paper we argue that MRI SPIONs, currently limited to major research centres, will have widespread clinical use in the future. We believe that knowledge about this growing area of research provides an opportunity for medical radiation practitioners to enhance their specialised expertise to ensure best practice in a truly multi-disciplinary environment. This review outlines how and why SPIONs can be synthesised and examines their characteristics and limitations in the context of MR imaging.

## INTRODUCTION

Nanotechnology has evolved into a multidisciplinary field, revolutionising industries such as applied physics, mechanical, chemical, electrical and biological engineering, machine design, robotics, and medicine [1]. In medical imaging, the development of nanoparticles has attracted a phenomenal amount of research, particularly for applications in molecular imaging.

The nano size (<100nm) of these particles enables conjugation with many molecular markers, which can interact at molecular and cellular levels, thereby offering an ever increasing range of disease targets for molecular imaging.

Nanoparticles also have the potential to revolutionise conventional imaging techniques [2]. Conventional imaging modalities lack the combination of high sensitivity and high spatial resolution required for molecular imaging. MRI has high resolution, but lacks sensitivity to molecular signals, while high sensitivity nuclear medicine modalities such as single photon emission computed tomography (SPECT) and positron emission tomography (PET) provide superb sensitivity, at the cost of reduced spatial resolution [2-4].

The use of nanoparticles in modalities like MRI can greatly increase sensitivity, presenting the potential for high-resolution molecular imaging. MRI has high spatial resolution [2, 5], is non-invasive in nature, uses non-ionising radiation, and offers multi-planar tomographic capabilities [2]. Nanoparticles can be engineered to have magnetic characteristics that can be detected by MRI at low concentrations, and at the same time contain ligands which target specific molecules [2].

Iron oxide nanoparticles have been widely researched for MRI, as they are mainly superparamagnetic. There are several types of iron oxide nanoparticles, namely maghemite, γ-Fe_2_O_3_, magnetite, Fe_3_O_4_, and haematite, α-Fe_2_O_3_, among which magnetite, Fe_3_O_4,_ is very promising, because of its proven biocompatibility [1].

For molecular imaging purposes, superparamagnetic iron oxide nanoparticles (SPIONS) need to be biocompatible, non-toxic and magnetic. They also need to bind to a range of drugs, proteins, enzymes, antibodies, or other molecular targets.

There have been a number of approaches to the production of SPIONS for use as MRI contrast agents, and each method produces particles with different sizes and magnetisation parameters. The iron oxide nanoparticles can also be coated with a surface layer, usually of organic material, that provides an interface between the core and the surrounding environment [6]. This surface layer can be used to direct the particles to a target site.

In this review, we summarise some of the chemical routes for the synthesis of SPIONS, such as classical synthesis, reactions in constrained environments, and high temperature reactions. It will also discuss some of the major methods for structural and physicochemical characterisation of the SPIONS, such as x-ray powdered diffraction (XRD), transmission electron microscopy (TEM), dynamic light scattering (DLS), nuclear magnetic resonance spectroscopy (NMR), and atomic absorption spectroscopy (AAS).

## SYNTHESIS OF IRON OXIDE NANOPARTICLES

### Nanoparticle design

Nanoparticles, being the smallest building block, can essentially be synthesised to have any structure, and can comprise a core and/or monolayer(s). For example, some drug delivery applications use multiple polymer layers surrounding an organic core [7], while some imaging applications use a basic structure that incorporates an inorganic core surrounded by an organic monolayer. The main materials used for the cores include metals such as gold (Au), platinum (Pt), silver (Ag), cobalt (Co), semiconductors cadmium selenide (CdSe), lead selenide (PbSe), or hybrids CdSe/zinc selenide(ZnS) [8].

Materials suitable for composing the organic monolayer can include; silica shells [12, 13], lipids [14-17], polymers [18, 19] and amphiphilic ligands [3, 9-11]. This layer can also be augmented with non-specific ligands or DNA fragments, antibodies, proteins, and drugs.

The choice of core and monolayer material is critical to the design of specialised contrast agents as each layer dictates a specific function. The composition of the core material dictates the primary physical and chemical properties of the nanoparticle, which in turn determine how it can be imaged. Iron particles, for example, are potentially very useful as MRI contrast agents because they are magnetic and behave as single magnetic domains when exposed to an external magnetic field. On the other hand, CdSe nanoparticles or ‘quantum dots’ can be used as optical probes for fluorescent imaging.

The monolayer provides the interface between the core and the surrounding environment [6] and can serve two purposes. Firstly, to act as a barrier between the nanoparticle core and the environment, to protect and stabilise the core [6]. Some materials used for the core such as iron oxides, on their own, are not stable, and are readily oxidised, changing valuable properties of the nanoparticle. Secondly, the chemical nature of monolayers dictate the reactivity, solubility and interfacial interactions [6], and may also determine the biological handling, of the nanoparticle. Most of the inorganic cores are not soluble in aqueous environments, and monolayer designs serve to overcome this problem, particularly for in-vivo applications. The inorganic core, when used alone, does not have a specific target, however if the monolayer is a particular molecular precursor or is conjugated to a specific molecule, it can direct the particle to an area of interest.

### Nanoparticle design for MRI

As well as having a suitable iron core and monolayer, SPIONS, need to possess a range of other properties to ensure they are useful as MRI contrast agents. These are:

- uniform particle size [20, 21]
- a uniform and high superparamagnetic moment [2, 20, 21]
- high colloidal stability [2]
- low toxicity and high biocompatibility [2]

The way SPIONs are produced has an influence on all of the above properties [2]. For MRI, these properties are important as they determine the overall effectiveness of the contrast agent. For example, an essential characteristic of an effective MRI contrast agent is a high saturation magnetisation value, (expressed in electromagnetic unit/gram, [emu/g]). Saturation magnetisation values are a measure of the magnetic moment, so higher values produce more magnetic susceptibility, and therefore stronger MRI signals [22].

Relaxation rates are a measure of the ability of a contrast agent to enhance the relaxation rate of water protons, i.e. increase the efficiency with which image contrast is produced [23]. SPIONS with high T2 values have faster relaxation with surrounding water protons, and therefore faster relaxation rates (1/T1 and 1/T2).

Typically, magnetisation values for SPIONS range from 30-50emu/g, while higher values such as 90emu/g have been observed for bulk material [24, 25]. Factors contributing to the magnetisation value of SPIONS include; the *size of the particles* (with the highest emu/g to volume ratio occurring in the 6-20nm particle size range [26]), *spacing between the nanoparticles* (where coatings such as silica separate the magnetic domains, allowing each individual magnetite particle to act independently and thus enhancing the net magnetism per gram) and the *crystalline structure* of the iron oxide. It is therefore essential to use a method of SPION production that generates particles with one or more of the above characteristics.

The overall size and size distribution of the SPIONS is an important consideration as it can affect the biocompatibility and biodistribution in-vivo. It is well known that particles above 50nm in diameter are eliminated by the reticulo-endothelial system (RES) so SPIONs greater than 50nm in diameter are limited to liver/spleen imaging. A range of synthesis methods have been developed to produce SPIONs with varying sizes and this relationship between size and biocompatibility will be discussed in the following section.

Other properties, such as high colloidal stability and low toxicity, are important, because they increase the chances of translating developmental contrast agents into the clinical setting.

The following sections will briefly discuss the basic method of SPION growth, and then discuss the different methods of SPION production and their respective properties for MRI.

### Nucleation and particle growth

In making iron oxide nanoparticles for MRI, the particles need to be of uniform size. Uniform particles are usually prepared via homogeneous precipitation reactions [2], which involve two processes, nucleation and growth. This is because iron oxide nanoparticles are crystalline structures that are governed by the principles of crystal formation and growth. Generally, for precipitation to occur, there must be a saturated solution, in which addition of any excess solute will cause precipitation, and the formation of nanocrystals [8].

For nucleation to occur, the solution must be supersaturated [2], leading to a short single burst of nucleation [27]. Supersaturation can be achieved by dissolving the solute at a high temperature, or by adding reactants to produce supersaturation [28]. After the short burst in nucleation, the concentration drops and nucleation stops. The nuclei then grow, by diffusion of solutes from the solution onto the nuclear surfaces, until an equilibrium concentration is achieved.

In order to achieve monodisperse particles, the two phases of nucleation and growth need to be separated [8, 20, 27, 29]. There are many different mechanisms which can explain this process, however we refer the reader to LaMer and Dinegar [30], who proposed the classical theory method of the formation of sulphur colloids, Den Ouden and Thompson who explained ‘Ostwald ripening growth’ [31, 32] and other mechanisms proposed by Morales *et al.* [33], and Ocana *et al.* [34].

Size control is ultimately achieved by artificially separating nucleation and growth. This would occur before the solution reaches critical supersaturation, or by the end of nucleation [20]. A wide variety of factors have been adjusted in many ways to promote separation of the two processes to control size, magnetic characteristics, or surface properties. Some of the factors have contributed to the development of new synthesis methods, and some have just improved classical methods. A few of these factors will be discussed below.

### Methods of superparamagnetic iron oxide nanoparticle synthesis

There are numerous methods of iron oxide nanoparticle synthesis for applications to MRI [20], for example; chemical precipitation, constrained environments and high temperature reactions. In keeping with the scope of this paper, only these selected methods will be discussed.

#### Chemical precipitation

The precipitation method is the simplest chemical pathway to obtain SPIONS [8, 20].

The SPIONS, either magnetite (Fe_3_O_4_), or maghemite (γFe_2_O_3_), are prepared by co-precipitating a stoichiometric mixture of ferrous and ferric salts in an aqueous medium. The thermodynamics of the reaction require a ratio of 2:1 for Fe^2+^/ Fe^3+,^ and a pH between 8 and 14. The precipitated magnetite is black in colour. The overall reaction can be written as [1, 20]:

(1) $${\text{Fe}}^{\text{2}+}+{\text{ 2Fe}}^{\text{3}+}+{\text{ 8OH}}^{-}\to {\text{ Fe}}_{\text{3}}{\text{O}}_{\text{4}}+{\text{ 4H}}_{\text{2}}\text{O}$$

The ions can become oxidised before precipitation, critically affecting the physical and chemical properties of the SPIONS. For iron oxide, or magnetite, oxidation usually means the formation of maghemite. The reaction must therefore be carried out under a nitrogen environment to eliminate oxidation.

The transformation from magnetite to maghemite can pose a serious problem for the production of contrast agents. The two differ from each other in the spinel structure; one occupies positions in the octahedral and tetrahedral sites, and the other, maghemite, has cationic vacancies in the octahedral position. This crystal structure results in a different net spontaneous magnetisation (or emu/g) of the iron particles : at 300^o^K, 92 emu/g^-1^ for magnetite, and 78 emu/g^-1^ for maghemite [35].

Most of the time it is difficult to separate magnetite from maghemite [36], given that their diffraction spectra are very similar [21]. Some synthesis methods suggest the presence of both magnetite and maghemite in the resulting preparations [37].

In the co-precipitation process there are two main processes involved. The first is a short single burst of nucleation, followed by growth of the nuclei, as discussed in the previous section. The precipitation method provides an advantage because large quantities can be synthesised; however, problems arise from the wide particle size distribution.

As mentioned above, size affects the magnetisation values as well as the biodistribution in-vivo. Factors that influence the biodistribution of a particle are important, as they also determine the possible MRI applications. To control the size, and size distribution, it is essential to adjust factors that determine the precipitation process. Numerous studies have been conducted adjusting factors such as pH, ionic strength, temperature, nature of salts, Fe^3+^/Fe^2+^ ratio, and addition of chelating agents, which improve the size and size distribution of the SPIONS produced.

The Massart process describes the co-precipitation of ferrous and ferric chlorides, and hydroxides in an alkaline solution [38]. Parameters such as strength of the base (eg ammonia or NaOH), the pH value, added cations, and the Fe^3+^/Fe^2+^ ratio were evaluated, noting the effect on yield of the co-precipitation reaction and particle sizes. It was concluded that the size decreases as the pH, and/or Fe^3+^/Fe^2+^ ratio, increase, and as ionic strength in the medium increases.

A comprehensive study on the ratio of Fe^2+^/Fe^3+^ was conducted by Jolivet *et al*. In 1992 [39] and 1994, illustrating the effects on size, morphology and magnetic characteristics. Small values of the Fe^2+^/Fe^3+^ ratio (<0.3) were known to form goethite. For ratios less than 0.5, but greater than 0.3, there were two phases, consisting of smaller (4nm) and larger nanoparticles. However, a ratio of 0.5 corresponded to magnetite stochiometry, and the particles were homogenous in size and composition.

In 1999, Babes *et al*. [40] investigated different properties such as iron concentration, temperature and oxygen. It was highlighted that one of the most important parameters was the Fe^2+^/Fe^3+^ molar ratio. A high ratio produced larger particles, which is consistent with the literature [39, 41], suggesting that only ratios between 0.4 and 0.6 produce monodisperse particles, suitable for use as contrast agents in MRI [20].

It is reported that the higher the pH and the ionic strength, the smaller the particle size and size distribution [41, 42]. Vayssières *et al*. [42] observed that for a higher pH and ionic strength, the particles were smaller due to the thermodynamics of the solution. At a lower pH and ionic strength, the particles continued to grow during the ageing phase associated with Ostwald ripening, thus forming larger particles.

A recent study on the size of the SPIONS, and its effect on magnetisation and MR signal, was conducted by Young-wook Jun *et al*. [26]. The SPIONS were highly crystalline, monodisperse, and stoichiometric for magnetite, and ranged in size from 4nm to 12nm in diameter. The general trend suggested that as the nanoparticles increased in size, the T2-weighted MR signal intensity decreased, the particles therefore appearing hypointense on T2-weighted images.

Apart from modulating the parameters of the reaction to achieve monodisperse particles, the addition, either in combination or individually, of chelating organic anions like citric acid [43, 44], amino acids, and dimercaptosuccinic acid (DMSA) [45], can also decrease the particle size by inhibiting the growth of the crystal nuclei. Polymer surface complexing agents, which form monolayers on the surface of the iron oxide, such as dextran [46], carbodextran, and silica [47] can also be added, instead of varying the reaction parameters.

Some polymer complexing agents such as dextran, carbodextran and silica are commercially available, and are currently used in iron oxide-based MRI contrast agents. Examples are: silica-coated magnetite, AMI-121 (Lumirem®- US) dextran-coated magnetite, Ferumoxides (Endorem® – Europe, Feridex® in the USA and Japan) and carboxydextran coated magnetite, Ferucarbotran (Resovist® – Europe and Japan).

It should be noted that these agents can be used for any method of iron oxide production. The coatings often serve multiple purposes; they allow for water solubility [2], the attachment of various functional probes [2, 48], promote the formation of monodisperse particles [20, 45] and stabilise the magnetite core [6].

Although the co-precipitation method is the simplest and most efficient chemical pathway to obtain magnetic particles, it has disadvantages such as large particle size distribution, aggregation and poor crystallinity, resulting in low saturation magnetisation values. These disadvantages have led to the development of advanced methods of magnetite synthesis [1].

#### Reactions in constrained environments

Synthesis reactions in constrained environments have made use of lipid-based structures with amphiphiles [12, 49-53] and dendrimers [54].

Lipid-based nanoparticles, or colloidal aggregates such as liposomes, micelles or microemulsions, are composed of lipids and/or other amphiphilic molecules. Amphiphiles (sometimes referred to as surfactants) are molecules with both hydrophilic (polar head) and hydrophobic (non-polar tail) parts that spontaneously assemble into aggregates in an aqueous solution [55]. Because of these properties, there are various geometries and sizes that can be formed due to unfavourable interactions between the hydrophobic tails and water [55], such as cylindrical, spherical, and bilayered.

The hydrophobic tails can vary in length, affecting the ratio between hydrophilic and hydrophobic parts, and the hydrophilic heads can also vary in charge and size, affecting the overall curvature of the aggregate. Other factors, such as pH, temperature and concentration, can also affect the end-product.

Mulder *et al.* [14] illustrate the various geometries that can be formed.

In micelle-forming lipids, the hydrophobic chains are oriented toward the inside of the micelle, and the hydrophilic chains outward. Micelles for MR imaging contain a hydrophobic core, where the iron oxide core is stabilised by the surfactant, which limits particle nucleation and growth [8].

The first magnetic nanoparticles formed in micelles were produced by oxidation of Fe^2+^ salts [56]. The size of the magnetite particles were controlled by varying the temperature and the surfactant concentration [57]. Micelles give control to the particle size formed, however reverse micelles are of importance for applications to MRI.

In reverse micelles, the hydrophilic head groups are towards the core of the micelle and the hydrophobic groups are directed outwards. Reverse micelles can solubilise relatively large amounts of water, which can be controlled, to make them suitable for the synthesis of nanoparticles. A diverse range of nanoparticles can be obtained by varying the nature and amount of surfactant, co-surfactant, and solvent.

Reverse micelles are essentially formed by aqueous iron salt solutions, encapsulated by a surfactant that separates them from the surrounding organic solution. Publications have suggested that iron oxide nanoparticles synthesised via the reverse micelle process can be used for MRI applications [58]. For example Lee *et al*. [57], investigated an inexpensive, large-scale, and highly crystalline method of magnetite production. The synthesis was carried out at high temperatures whilst varying the relative proportion of iron salts, surfactant and solvents. It was suggested that the particle size could be controlled to produce monodisperse particles in one sample.

Poly(ethylene glycol) (PEG) stabilised lipids can also be used for targeting and stabilising the iron oxide core [59]. The advantages of using PEG stabilised lipids are long blood circulation times, and water solubility, while the disadvantages are associated with difficult preparation methods, and excessive size separation processes [60].

Bi-layer forming lipids are used to create liposomes; they usually have a polar head group and two fatty acid chains. Iron oxides can be placed inside the liposomal lumen to create magnetoliposomes [61]. There are two types of magnetoliposomes; the first consists of water-soluble iron oxide particles within an aqueous lumen [62]. The second contains iron oxide particles of approximately 15nm, covered with a lipid bi-layer [63].

The second type, developed by De Cuyper and Joniau [63], has been used in-vivo for MRI as a bone marrow contrast agent [15]. The magnetoliposomes are produced by first synthesising iron oxides in solution. The particles are then solubilised and stabilised by the addition of laurate, which acts as a surfactant. A solution with excess phospholipids is then added to the particles and undergoes dialysis for a number of days. The surfactant molecules on the iron oxide surface exchange with the phospholipid molecules which, over time, cause the formation of a lipid bi-layer on the iron oxides nanoparticles. Furthermore, molecules such as PEG can also be added to the lipid bi-layer, increasing the half life in blood [64] and therefore increasing the number of applications for MRI contrast.

Dendrimers are a class of transfection agents that contain three components: core, branches and end-groups. When dendrimers are coated to iron oxides they are termed magnetodendrimers. Carboxylated polyamidoamine dendrimers have been used to coat and stabilise the iron oxide nanoparticles [54, 65]. More importantly, magnetodendrimers are well suited for the imaging of cell trafficking and migration using MRI [66-68]. This is due to the charge on the polymer, which promotes a high non-specific affinity for cellular membranes, resulting in cellular internalisation [65, 67].

Generally, the oxidation of Fe(II) at an elevated temperature and pH, in the presence of dendrimers, results in the formation of highly stable and soluble SPIONS with dendrimers [54]. They have an approximate size of 20-30nm, and high T2 relaxivities [54]. Cells from different origins: mouse, rat or human, can then be easily labelled to the magnetodendrimers, by introducing the magnetodendrimers to the cell culture for 1-2 days at low concentrations [66].

#### High temperature methods

Monodisperse particles with significant size control, and high crystallinity, can be achieved using high temperature methods. In this method, iron complexes are decomposed in the presence of surfactants and organic solvents. The high temperatures used in this method, and the nature of the solvent, result in the SPIONS having suitable size, and size distribution, with high crystallinity [69].

There are many studies on the synthesis of SPIONS using the high temperature method, for example Sun and Zeng [70] prepared iron oxide nanoparticles of different sizes, 3nm to 20nm. In this reaction, iron(III) acetylacetonate was decomposed by heating at 265^o^C in phenyl ether, alcohol, oleic acid, and oleylamine, to produce SPIONS 4nm in diameter. To make larger particles, a seed-mediated growth was used, controlling the quantity of seeds added to obtain various sizes.

Similarly, Hyeon *et al*. [71] formed an iron oleate complex from the decomposition of iron pentacarbonyl in the presence of octyl ether and oleic acid at 100^o^C. After cooling to room temperature, (CH3)3NO was added, and then the SPIONS were obtained by heating, followed by refluxing. When the molar ratios of iron pentacarbonyl and oleic acid were changed from 1:2 to 1:4, the particle size increased from 7nm to 11nm.

In another study by Park *et al.* [72], iron salts were used instead of toxic organometallic compounds such as iron carbonyl. Iron salts are more suited for contrast agent research and applications in MRI because they are less toxic. An iron-oleic complex was formed using iron chlorides, (FeCl3·6H2O) and sodium oleate, which was slowly heated to 320^o^C in 1-octadecene. The solution was aged at this temperature for 30 minutes, generating monodisperse iron oxide crystals. Various temperatures and solvents were also tried, which produced particles of different sizes and dispersity. It was concluded that monodisperse particles could be attributed to the separation of growth and nucleation phases, which occured at different temperatures; nucleation at 200-240°C, and growth at 300°C.

### Monolayers for superparamagnetic iron oxide nanoparticles

On their own, iron oxides are not very stable, and are not soluble in water. Stabilisation of SPIONS is essential to prevent against aggregation and oxidisation. Furthermore, for use as MRI contrast agents in-vivo, the SPIONS need to be soluble in water and be easily conjugated to molecular and cellular markers.

As discussed briefly in the previous sections, there are numerous ways for SPIONS to achieve water solubility and stability. Some of these methods include coating with carboxylates (such as citric acid), inorganic materials such as silica, and polymers such as dextran and PEG. These compounds protect the iron core, and also provide an avenue for conjugation of molecular precursors, therefore providing a biocompatible functional component for the SPIONS.

#### Carboxylates

The surface of the magnetite nanoparticles can be stabilised in an aqueous dispersion by the absorption of citric acid [72]. This process, as described in Sahoo *et al*. [45], occurs by the citric acid being coordinated via one or two of the carboxylate functionalities, depending on steric necessity, and the curvature of the surface. As a result, at least one carboxylic acid group is exposed to the solvent, and this group is responsible for making the surface charged and hydrophilic. The presence of the terminal carboxylic group provides an avenue to extended bond formation with fluorescent dyes, proteins, hormone linkers, and other molecules, so that specific targeting within biological systems can be facilitated.

Molecules such as DMSA can also be used to stabilise the SPIONS, achieve water solubility and allow conjugation of molecular precursors [73]. DMSA has successfully been used as a monolayer [74], where the DMSA is introduced to the SPIONS, in excess, through simple mixing. The DMSA binds to the magnetite surface through its carboxylate bonding, and the intermolecular disulfide cross-linking between surface-bound DMSA ligands strengthens the stability. The remaining free carboxylic acid and thiol groups make the SPIONS hydrophilic, and can be used for further conjugation of target-specific antibodies.

#### Silica

Iron oxide nanoparticles can also be coated with silica [74]. Silica is an inert molecule that coats the surface of the iron oxide nanoparticle, and, as a result, prevents aggregation of the SPIONS, and provides stability [75]. This is achieved by two processes: (1) sheltering of the magnetic dipole interaction by the silica shell; and (2) charging the magnetic nanoparticles, as silica is negatively charged [47]. These two features are essential, particularly for applications in MRI, as aggregation of the magnetite particles can reduce or diminish their ability to be superparamagnetic [76].

There are two widely used methods to produce silica-coated iron oxide nanoparticles. The first method is based on the Stober process [76], which comprises the hydrolysis and condensation of a sol-gel precursor such as tetraethyl orthosilicate (TEOS). There have been numerous studies conducted on the formation of iron oxides coated with silica using the Stober process [46, 77].

The second most common method of generating iron oxide-coated silica nanoparticles is via the microemulsion process, where reverse micelles are used to confine and control the silica coating. In this method, non-ionic surfactants are used to form inverse microemulsions for preparation or suspension of magnetic nanoparticles [78]. The silica is formed around the magnetic nanoparticles by hydrolysis and condensation of TEOS [79].

#### Dextran

Dextran is a polysaccharide polymer that is composed of α-D-glucopyranosyl units and can vary in length (1000 to 2,000,000 Da) and branching. Dextran offers a suitable monolayer for SPIONS because of its biocompatibility [80]. The formation of iron oxide coated by dextran was first documented by Molday and Mackenzie [80]. In this study, dextran 40 000 was coated to the iron oxide nanoparticles by reacting a mixture of ferrous chloride and ferric chloride with the dextran polymers, under alkaline conditions.

Other studies have looked at smaller dextran coatings such as dextran 10 000 [21, 81, 82]. Reducing the size of dextran has an effect on the formation and stability of the dextran-coated iron oxide nanoparticles [83, 84]. Paul *et a*l. [85] describe that the smaller dextran has significant effects on particle size, coating stability, and magnetic properties. It was concluded that SPIONS coated with a reduced dextran were more stable than those coated with a larger molecular weight dextran. Higher molecular weight dextran produced larger particles, and only the 10,000 Da dextran gave a particle with high magnetic properties.

### Characterisation of superparamagnetic iron oxide nanoparticles

There is a wide variety of analysis tools to characterise SPIONS. It is important to define the exact characteristics of SPIONS, as these characteristics can influence the application of SPIONS in MRI.

For any biological application, a range of tests such as biocompatibility, toxicity and efficacy, needs to be considered. However, within the scope of this paper, and for preliminary development of SPIONS in MRI, the most general properties that need to be analysed are the physical (size, shape, chemical phases) and magnetic (MR properties, magnetic saturation values (emu/g)) properties.

#### Physical properties

When analysing the size of the SPIONS, we are in fact measuring a range of dimensions. This includes different parts of the nanoparticle: size of the iron oxide core, size of the monolayer e.g. silica or DMSA, size of the iron oxide core and monolayer, e.g. silica or DMSA, combined. It also includes the size range of the particles present in the sample.

The size of the iron oxide core can be determined by transmission electron microscopy (TEM) [83-85]. TEM gives the total particle size, core and monolayer, and also provides details on the size distribution and the shape of the SPIONS. There are two different types of TEM; low resolution and high resolution. With the high resolution TEM, the atomic arrangement of the SPIONS can be deduced. It also allows better characterisation, or separation, between the core and monolayer. The lattice arrangement and the surface atomic arrangement of the crystals can also be studied, by the use of diffraction patterns.

Generally for TEM, a small portion of the sample is placed on a coated copper grid and then imaged. Although it provides precise direct information about size, size distribution and shape of the particles, it has several disadvantages such as operator bias, a risk of change in particle properties as the sample dries and contrasting of the sample [21].

Dynamic light scattering (DLS) is also a useful technique in particle size characterisation, and holds some advantages over TEM. DLS can obtain information about the size and size distribution in solutions generally at a lower cost and with less time. In DLS, the distribution of diffusion coefficients are calculated which are transformed into measurements of the hydrodynamic or total diameter of the particles [21, 86]. Like all modalities, DLS also has disadvantages such as contamination by dust or small amounts of aggregates in the sample; these can create misleading results [21].

X-ray powdered diffraction (XRD) can also be used to estimate the size of the particles, and the crystalline structure. XRD gives a diffraction pattern of the sample, and this is compared to a reference peak or pattern. Line broadening from the XRD pattern is used to calculate the crystal sizes [87], using the Scherrer formula, and these results can indeed be compared to the TEM results.

Mossbauer spectroscopy is another method that can be used to approximate the size of the SPIONS [87] to complement DLS and TEM results. A resonant absorption of nuclear gamma radiation, e.g. of the non-radioactive ^57^Fe isotopes, gives information on the magnetic coupling of the sample, the valence state of the iron ions, and also on the size of the core.

#### Magnetic properties

Measurements of the magnetisation as a function of the applied magnetic field allow the determination of magnetic properties: magnetic susceptibility, saturation magnetisation, and r_1_ and r_2_ relaxivities.

A vibrating sample magnetometer (VSM) or Superconducting Quantum Interference Device (SQUID) magnetometer can be used to analyse the magnetic properties. Parameters such as magnetic moment and hysteresis loop measurements can be measured. The necessary equipment is scarce, and although these parameters are useful, for the requirements of MRI, other analytical tools can be used.

Nuclear magnetic resonance spectroscopy (NMR) can be used to analyse properties such as the T_1_ and T_2_ relaxivities. After analysing the iron concentrations (see below), the r_1_ and r_2_ relaxivities can be calculated by plotting the T_1_ and T_2_ over the iron concentration [88]. Alternatively, MRI can be used, using the same process for analysis.

#### Iron concentration

The iron concentration of the sample is generally measured using atomic absorption spectroscopy (AAS), or inductively coupled plasma mass spectrometry (ICPMS).

AAS compares light absorbance of the unknown sample with light absorbance from known calibrated standards. It is relatively easy to use and well understood; however some limitations are that only one sample can be measured at a time and there can be interference with some elements. On the other hand, ICPMS is a form of mass spectrometry that is highly sensitive and can determine a range of metals and non-metals at very low concentrations.

### MRI with SPIONS: current status and future directions

The previous sections discuss many studies that have researched and developed SPIONS. Some research has improved magnetic characteristics, while other studies have developed novel methods for reducing the size of the SPIONS, as well as producing monodisperse particles. Despite research over many years, SPIONS as MRI contrast agents, particularly for molecular imaging, are still not available clinically. The problems in the translation of SPION research for MRI to the clinic lie primarily in particle size: larger particles limit applications; dosage: large amounts of SPIONS are needed to produce adequate contrast; and production: the ability to adapt synthesis methods to industrial levels of production.

SPIONS that are larger than 50nm are eliminated by the RES, therefore they are mainly useful for liver/spleen imaging applications. Smaller SPIONS are also taken up by the RES; however, because of their smaller size, their blood circulation time is longer, providing greater opportunity for specific localisation. SPIONS produced commercially generally have had narrow applications and have been withdrawn from some markets due to low demand. An example is SHU-55A, Resovist. Resovist has an iron oxide core of 4.3nm and is coated with carbodextran to a total diameter of 60nm. Resovist also has excellent T2 relaxivities of 151.0 mmol^-1^sec -^1^, and was used only for liver/spleen imaging. Products like OMP (Abdoscan) and AMI-121 (Lumirem, Gastromark) have total diameters of approximately 300nm and are coated with polystyrene and silica. They are administered orally and used for gastro-intestinal contrast. Unfortunately the prices of these products are high, and they are only available on the European and US markets.

Smaller-sized SPIONS such as AMI-227 (Sinerem, Combidex) and SHU-55C (Supravist an optimisation of Resovist, SHU-55a) are ultra-small iron oxide particles coated with dextran and carboxy-dextran respectively. Both products yielded a total diameter of approximately 20nm.They have been proposed for applications in lymph node and bone marrow imaging, as well as the imaging of inflammatory processes. These products are not yet approved and are still undergoing development and/or clinical trials. Another product, NC100150 (Clariscan) for perfusion/MR angiography was discontinued. The iron core was 5-7nm, and it was coated with PEG, having a total diameter of 20nm.

There are other SPION contrast agents that are available at a pre-clinical imaging level. Monocrystalline iron oxide particles (MION) are used for angiography, lymphography, tumour detection, and infarction. Companies such as BioPhysics Assay Laboratory (BioPAL) Inc. provide products such as Molday ION and FerroTrack that can be used for molecular and cellular imaging at the pre-clinical level. See Table 1 for a summary of SPION contrast agents.

**Table 1:** Summary of SPION Contrast Agents

Name		Status	Application	Administration	Relaxivites mmol^-1^sec^-1^	Coating	Core size (nm)
AMI-121	Lumirem and Gastromark	US and Europe	GI	Oral	T2 - 72 T1 - 3.2	Silica	300
OMP	Abdoscan	Europe	GI	Oral	N/A	Poly–styrene	300
AMI-25	Endorem and Feridex	US	Liver / spleen	IV	T2- 98.3 T1- 23.9	Dextran	5.6
SHU-55A	Resovist	Withdrawn from some markets	Liver / spleen	IV	T2 - 151.0 T1 - 25.4	Carbo–Dextran	4.2
AMI-227	Sinerem and Combidex	Clinical trial	Lymph node Bone marrow	IV	T2 - 44.1 T1 - 21.6	Dextran	4-6
NC100150	Clariscan	Discontinued	Perfusion / angiography	IV	T2- 35 T1- 20	Carbo–hydrate-PEG	5-7
SHU-55C	Supravist	Preclinical	Perfusion Lymph node Bone Marrow	IV	T2 - 57 T1 -7.3	Carbo–Dextran	3-5
MION 46		Preclinical	Angiography Lymph node Tumour Infarction	IV	N/A	Dextran	4-6
BioPal		N/A	Animal Imaging only	IV	Various	Various	N/A

For SPIONS to be used for more than one application, they need to be coated with a monolayer that can promote the attachment of molecular and cellular probes. An example is a study by Hilger *et al*. [88], in which iron oxide nanoparticles were coated with dextran, and then attached to anti-Her2/neu antibodies via the carboxyl groups on the dextran surface, for breast cancer imaging. Most of the SPIONS available or undergoing developments (see Table 1) have suitable surface coatings for molecular/cellular imaging; however, their clinical and/or proposed uses have been restricted to relatively narrow generic roles.

Another drawback in the translation of contrast agent research to the clinic has been the large amount of iron needed to produce adequate contrast. The challenge is to develop highly magnetic particles that can produce the strong signal enhancement, allowing low doses of SPION to be administered without compromising the MR signal.

Other problems are in translating the synthesis of SPIONS, easily made in the lab, to industrial processes able to produce large quantities on a consistent basis.

With the wide range of SPIONS that are currently being developed for single MR applications there are possibilities that in the future these SPIONS will be available for use as MR contrast agents. As for molecular and cellular imaging with MRI, the current research sets a platform for the further development of SPIONS. If SPIONS as MR contrast agents for single applications can be utilised, then the next step in SPION development would be towards molecular imaging. Although molecular imaging with MRI will not likely replace nuclear medicine and PET, it may play a useful complementary role. The current decade has seen extensive progress in SPION design, utilisation and characteristics, and we expect that the future will see highly magnetic SPIONS available for molecular and cellular imaging in MRI.
